# Salivary and Plasmatic Antioxidant Profile following Continuous, Resistance, and High-Intensity Interval Exercise: Preliminary Study

**DOI:** 10.1155/2019/5425021

**Published:** 2019-11-26

**Authors:** Adriele V. Souza, Jéssica S. Giolo, Renata R. Teixeira, Danielle D. Vilela, Leonardo G. Peixoto, Allisson B. Justino, Douglas C. Caixeta, Guilherme M. Puga, Foued S. Espindola

**Affiliations:** ^1^Institute of Biotechnology, Federal University of Uberlandia, Minas Gerais, Brazil; ^2^Faculty of Physical Education and Physiotherapy, Federal University of Uberlandia, Minas Gerais, Brazil

## Abstract

The increase in antioxidant responses promoted by regular physical activity is strongly associated with the attenuation of chronic oxidative stress and physiological mechanisms related to exercise adaptation. The aim of this work was to evaluate and compare how different exercise protocols (HIIE: high-intensity interval exercise, CE: continuous exercise, and RE: resistance exercise) may alter salivary and plasmatic antioxidants and salivary markers of exercise intensity and nitric oxide. Thirteen healthy, trained male subjects were submitted to the three exercise protocols. Blood and saliva samples were collected at the points preexercise, postexercise, and 3 hours postexercise. Antioxidants (total antioxidant capacity, superoxide dismutase and catalase activities, and levels of reduced glutathione and uric acid), markers of exercise intensity (salivary total protein and amylase activity), and salivary nitric oxide were evaluated. As a result, all exercise protocols increased the markers of exercise intensity and nitric oxide. Antioxidant response was increased after exercise, and it was found that a single HIIE session exerts a similar pattern of antioxidant response compared to CE, in plasma and saliva samples, while RE presented minor alterations. We suggest that HIIE may lead to alterations in antioxidants and consequently to the physiological processes related to redox, similar to the CE, with the advantage of being performed in a shorter time. In addition, the antioxidant profile of saliva samples showed to be very similar to that of plasma, suggesting that saliva may be an alternative and noninvasive tool in sports medicine for the study of antioxidants in different physical exercise protocols.

## 1. Introduction

The practice of physical exercise is reported to be associated with the production of reactive oxygen species (ROS). To neutralize these molecules and to attenuate the damage promoted by oxidative stress, the organism has an antioxidant system with enzymatic and nonenzymatic mechanisms, including the enzymes superoxide dismutase (SOD) and catalase (CAT), the glutathione system, uric acid, and also vitamins and minerals that act as cofactors for different antioxidant enzymes [1, 2].

Despite the deleterious effects of oxidative stress, ROS production is fundamental for several physiological mechanisms associated with exercise adaptation, such as cell signaling that stimulates processes of mitochondrial biogenesis and increase in muscle oxidative capacity and that promotes increased antioxidant efficiency [3, 4]. The regular practice of physical exercises then induces an adaptive process in the body, where the constant production of ROS stimulates the increase in enzymatic and nonenzymatic antioxidants, leading physically trained individuals to be less susceptible to chronic oxidative damage [5].

Aerobic exercises can induce oxidative stress and consequently induce the increase in antioxidant defense, mainly due to the increase in oxygen consumption levels, which will lead to a greater release of superoxide anions by the electron transport chain. Although anaerobic exercise involves less oxygen circulation, it can also generate ROS increase by different mechanisms and pathways, such as the activation of xanthine oxidase and nicotinamide adenine dinucleotide, ischemia-reperfusion injury, increased purine catabolism, disruption of Ca^2+^ homeostasis, and autooxidation of catecholamines [6, 7].

Among the different training modalities, high-intensity interval training (HIIT) has been shown to be an alternative protocol to traditional training, since it promotes improvements in both aerobic and anaerobic fitness [8]. HIIT can be defined as the practice of exercises characterized by brief outbursts of vigorous activity, interspersed with periods of rest or low-intensity exercise [9], and its practice has gained attention since it comprises a training method that can be performed in a short period of time, which improves adherence to training [10]. Evidence also suggests that HIIT is a more enjoyable training method to be practiced when compared to a continuous exercise of moderate intensity [11].

To our knowledge, there is no study that evaluated antioxidants following high-intensity interval exercise in salivary samples, and this biological fluid is also poorly explored in other exercise modalities in healthy individuals [12–14]. Saliva represents an interesting tool for the evaluation of biomarkers in the field of sports medicine, mainly because of the ease in the collection procedure, which is not invasive, does not require a qualified professional to perform, and does not cause pain or discomfort, as occurs in blood collections [15]. Thus, several analyses in salivary samples have been studied in the exercise area, such as for the monitoring of chronic stress markers like cortisol and testosterone hormones [16] and endothelial function, through analysis of salivary nitric oxide levels [17] and exercise intensity, such as salivary lactate, amylase activity, and total proteinconcentration. Salivary alpha-amylase can be considered a biomarker of exercise intensity, since its activity correlates with the lactate threshold. In the same way, salivary protein concentration can also indicate an anaerobic threshold, since it correlates with blood lactate in effort tests [18, 19].

Some evidence suggests that exercise may affect antioxidants and other oxidative stress markers in a variable way, according to variations in the type (if it is aerobic or anaerobic), duration (longer or shorter), and the intensity of the exercise applied (higher or lower) [20–22]. Thus, the aim of this work was to evaluate and compare how different exercise protocols applied to trained individuals (high-intensity interval exercise, continuous exercise, and resistance exercise) may alter salivary and plasmatic antioxidants and also salivary markers of exercise intensity and nitric oxide.

## 2. Material and Methods

### 2.1. Subjects

This study was performed with thirteen trained healthy males (volunteers) (age: 27.62 ± 1.28 years, height: 174.20 ± 2.14 cm, weight: 72.07 ± 1.77 kg, BMI: 23.76 ± 0.54 kg m^−2^, and body fat: 15.16 ± 0.99%; mean ± SEM), who had no oral inflammatory diseases and were nonsmoking and not taking regular or incidental medication or antioxidant supplements. Volunteers reported to perform both aerobic and anaerobic exercises at least 3 times a week in the last 6 months.

All experimental procedures were carried out in accordance with the Code of Ethics of the World Medical Association (Declaration of Helsinki) and were approved by the Institutional Review Board of the Federal University of Uberlandia (no. 1.908.151). The subjects were given informational briefings, and they provided voluntary, written informed consent for participation.

### 2.2. Experimental Procedures

Subjects performed in a randomized crossover fashion design three exercise sessions on separate occasions, separated by a minimum of 72 hours: high-intensity interval exercise (HIIE), continuous exercise (CE), and resistance exercise (RE).

The evaluation of the aerobic capacity of the participants was performed using the maximal aerobic test in a mechanically braked cycle ergometer (Cefise, Campinas, SP) with an initial load of 100 W and an increment of 45 W every 2 minutes. The entire test was performed with a rotation of 90 rpm. During the test, heart rate (Polar RS800, Finland) and perceived exertion were evaluated using the Borg score scale [23]. The criterion of interruption of the test was a perceived exertion scale score of 20 associated with inability to maintain rotation at 90 rpm and voluntary withdrawal due to exhaustion. The intensity associated with VO_2max_ (wVO_2max_) was considered the last complete stage of the incremental test.

The 12-repetition maximum (12-RM) test was performed to obtain the maximum load used in the RE protocol. A maximum of 5 attempts per apparatus were performed, and the maximum load of each exercise was determined in the same sequence as the exercises performed in the acute experimental session.

The high-intensity interval exercise (HIIE) protocol was performed in a cycle ergometer (Cefise, Campinas, SP), consisting of 1 min cycling bouts at 100% of wVO_2max_, interspersed with 1 min of passive recovery periods at 40% of VO_2max_ until voluntary exhaustion. The continuous exercise (CE) protocol consisted of continuous cycling for 60 min at 50-60% of wVO_2max_. Both aerobic exercises were performed with a rotation of 90 rpm. The resistance exercise (RE) protocol was performed with 3 sets of 12-repetition maximum (12-RM) in squat (smith machine), leg press 45°, lying leg curl, and stiff exercises, in that order, with a 2 min recovery interval between sets and exercises. For the squat, feet were placed parallel, aligned with shoulders, and the malleolus was placed over a marker on the ground. A manual goniometer was used to determine the squat depth in 90° (±5°) knee angle. The lowest point of the bar height for the squat depth was marked at the smith machine for each participant.

The participants were instructed to follow normal diet and to avoid physical activity and refrain from consuming alcohol and caffeinated beverages for 24 h prior to each exercise session. Volunteers were also instructed to have breakfast one hour before the exercise session and to maintain the same diet for the breakfast in the subsequent experimental sessions. A standard snack was provided to the subjects just after exercise. After that, the subjects stayed in fasting condition until the last collection. All protocols were performed in the morning.

### 2.3. Sample Collection

Blood from the antecubital vein (approximately 4 mL per collection) was collected from the subjects and placed in EDTA-coated tubes by a qualified phlebotomist using standardized venipuncture techniques. Unstimulated saliva samples were collected in plastic tubes by a spitting method. Volunteers rinsed their mouth with water, and they were instructed to swallow the remaining water in the oral cavity and to wait a minute before saliva collection. All collection procedures were performed in the morning, at the following moments: at rest, immediately after the exercise session, and 3 hours after. These time points were selected in order to verify the effects promoted right after exercise and also to evaluate the recovery time of these biomarkers.

To determine the salivary flow, the initial weight of the tubes was discounted from their weight after collection. The resulting weight was divided by the total collection time (3 minutes). Blood and saliva samples were centrifuged at 3000 rpm at 4°C for 20 minutes, and the supernatant was aliquoted. All samples were kept frozen at -80°C until analysis. All biochemical determinations were done in duplicate.

#### 2.3.1. Determination of Salivary Biomarkers of Exercise Intensity

*(1) Alpha-Amylase Activity*. The saliva samples were diluted in MES buffer (50 mM MES, 300 mM NaCl, 5 mM CaCl_2_, and 140 mM KSCN; pH 6.3) and pipetted into a microplate, then preheated (37°C) substrate solution (2-chloro-4-nitrophenyl-*β*-D-galactopyranosylmaltoside (GALG2-CNP)) was added. The optical density was read at 405 nm in 1 min intervals for three minutes at 37°C using a microplate reader, then the enzyme activity was determined [17].

*(2) Total Protein Quantification*. Salivary total protein quantification was performed by the Bradford method [24], and results were calculated using a bovine serum albumin curve.

#### 2.3.2. Salivary Nitric Oxide (NO)

Nitrite concentration, an indicator of the production of nitric oxide, was determined by a colorimetric assay using the Griess reaction. Equal volumes of saliva and Griess reagent (1% sulfanilamide and 0.1% N-(1-naphthyl) ethylenediamine dihydrochloride in 2.5% phosphoric acid) were mixed at room temperature. Absorbance was measured at 570 nm using a microplate reader. The content of nitrite was calculated based on a standard curve constructed using sodium nitrite (NaNO_2_) [17].

#### 2.3.3. Determination of Antioxidants in Saliva and Plasma Samples

*(1) Total Antioxidant Capacity by Ferric-Reducing Antioxidant Power (FRAP) Analysis*. Total antioxidant potential was determined by the capacity of the antioxidants, present in plasma and saliva samples, to reduce Fe^+3^ to Fe^+2^ that is chelated by TPTZ (2,4,6-tris(2-pyridyl)-s-triazine) and form the complex Fe^+2^-TPTZ. This colored complex was read in the spectrophotometer at 593 nm, and the antioxidant activity was determined using an analytical curve, constructed with trolox as a standard [25].

*(2) Enzymatic Antioxidants: Superoxide Dismutase (SOD) and Catalase (CAT) Activities*. SOD activity was assessed by the inhibition of autooxidation of pyrogallol by SOD present in the samples. Samples were mixed with 50 mmol L^−1^ Tris-HCl buffer (pH 8.2) containing 1 mmol L^−1^ EDTA to deactivate metal-dependent enzymes such as metalloproteases, 80 U mL^−1^ catalase, and 24 mmol L^−1^ pyrogallol, and the kinetic assay was monitored for 10 minutes at 420 nm using an analytical curve constructed with SOD as the standard [26].

CAT activity evaluation was based upon hydrogen peroxide decomposition by CAT present in the samples. Samples were mixed with 10 mmol L^−1^ potassium phosphate buffer (pH 7.0) containing 0.2% hydrogen peroxide. The hydrogen peroxide decomposition was monitored at 240 nm for 10 min [26].

*(3) Nonenzymatic Antioxidants: Reduced Glutathione (GSH) and Uric Acid Levels*. For the quantification of GSH levels, 150 *μ*L of sample was added to 150 *μ*L of metaphosphoric acid and then centrifuged at 7000 × *g* for 10 min at 4°C. 30 *μ*L of the supernatant was mixed with 185 *μ*L of 100 mM sodium phosphate buffer (pH 8.0) containing 5 mM EDTA and 15 *μ*L of ortho-phthaldialdehyde (1 mg/mL in methanol). The solution was incubated in the dark at room temperature for 15 min. Fluorescence was read at 350 nm (excitation) and 420 nm (emission). Sample concentrations of GSH were calculated using a standard GSH curve (0.001-0.1 mM) [25].

Uric acid levels were evaluated using the enzymatic colorimetric method according to the recommendations of the manufacturer of the Labtest® kit.

### 2.4. Statistical Analysis

Data normality was determined using the Shapiro-Wilk test and analyzed using a one-way ANOVA with repeated measures followed by Tukey's posttest. Correlation analysis was performed using Pearson's correlation method. All data are reported as mean ± SEM, and statistical analysis was conducted at the 95% level of significance (*p* ≤ 0.05). Analyses were performed in the GraphPad Prism program (GraphPad Prism version 7.0 for Windows; GraphPad Software, San Diego, CA, USA).

## 3. Results

Table 1 shows salivary flow in all exercise protocols at preexercise (pre-ex), postexercise (post-ex), and three hours postexercise (3 h post-ex). No differences were observed at post-ex compared to pre-ex. 3 h post-ex was verified to have an increase in salivary flow when compared to post-ex in the HIIE and CE.

**Figure 1:**
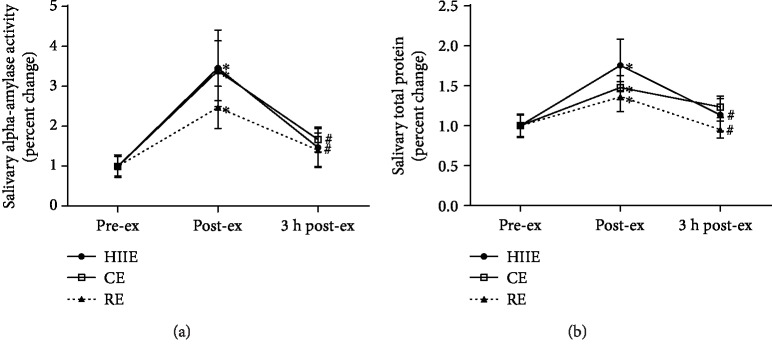
Biomarkers of exercise intensity. Salivary alpha-amylase activity (a) and salivary total protein (b) at preexercise, postexercise, and 3 hours postexercise. HIIE: high-intensity interval exercise; CE: continuous exercise; RE: resistance exercise. Values expressed as mean percentage in relation to preexercise ± SEM. ^∗^*p* ≤ 0.05 vs. pre-ex; ^#^*p* ≤ 0.05 vs. post-ex. ANOVA-RM followed by Tukey's test (*n* = 13).

Figure 1 shows the salivary biomarkers of exercise intensity. The activity of salivary alpha-amylase increased in all exercise protocols at post-ex compared to pre-ex. At 3 h post-ex, the enzyme activity decreased in both HIIE and CE when compared to that at post-ex (Figure 1(a)). In the same way, salivary total protein concentration also increased in all exercise protocols at post-ex compared to pre-ex. At 3 h post-ex, protein content decreased in both HIIE and RE (Figure 1(b)).

**Figure 2:**
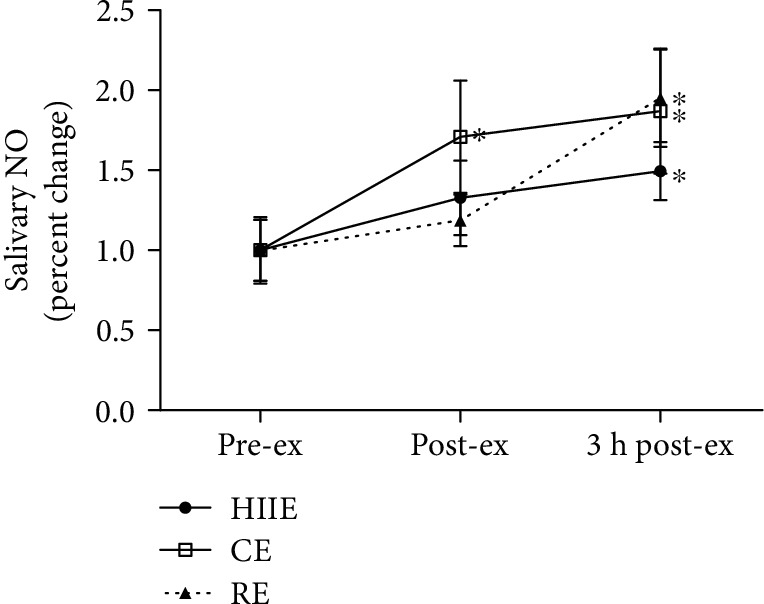
Salivary nitric oxide (NO) at preexercise, postexercise, and 3 hours postexercise. HIIE: high-intensity interval exercise; CE: continuous exercise; RE: resistance exercise. Values expressed as mean percentage in relation to preexercise ± SEM. ^∗^*p* ≤ 0.05 vs. pre-ex. ANOVA-RM followed by Tukey's test (*n* = 13).

Regarding NO concentration, an increase was observed in CE at post-ex compared to pre-ex. Interestingly, at 3 h post-ex, there was an increase in salivary NO levels in HIIE and RE compared to those at pre-ex, whereas CE remained increased (Figure 2).

**Figure 3:**
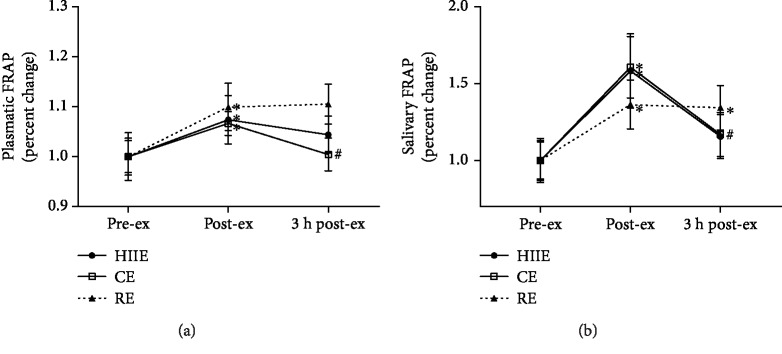
Total antioxidant capacity by ferric-reducing antioxidant power (FRAP) analysis in plasma (a) and saliva (b) at preexercise, postexercise, and 3 hours postexercise. HIIE: high-intensity interval exercise; CE: continuous exercise; RE: resistance exercise. Values expressed as mean percentage in relation to preexercise ± SEM. ^∗^*p* ≤ 0.05 vs. pre-ex; ^#^*p* ≤ 0.05 vs. post-ex. ANOVA-RM followed by Tukey's test (*n* = 13).

**Figure 4:**
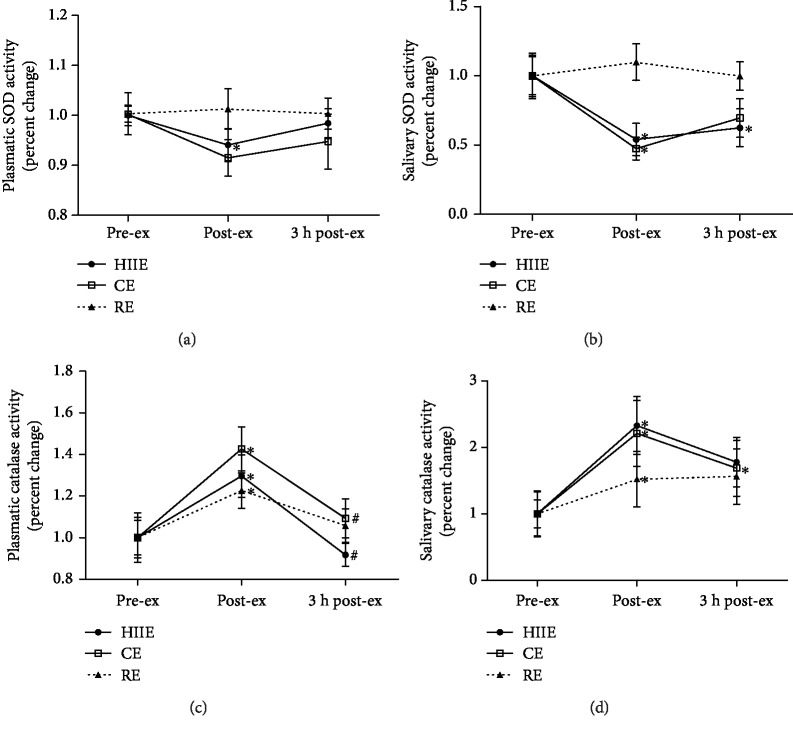
Enzymatic antioxidants: superoxide dismutase (SOD) activity in plasma (a) and saliva (b); catalase (CAT) activity in plasma (c) and saliva (d), at preexercise, postexercise, and 3 hours postexercise. HIIE: high-intensity interval exercise; CE: continuous exercise; RE: resistance exercise. Values expressed as mean percentage in relation to preexercise ± SEM. ^∗^*p* ≤ 0.05 vs. pre-ex; ^#^*p* ≤ 0.05 vs. post-ex. ANOVA-RM followed by Tukey's test (*n* = 13).

**Figure 5:**
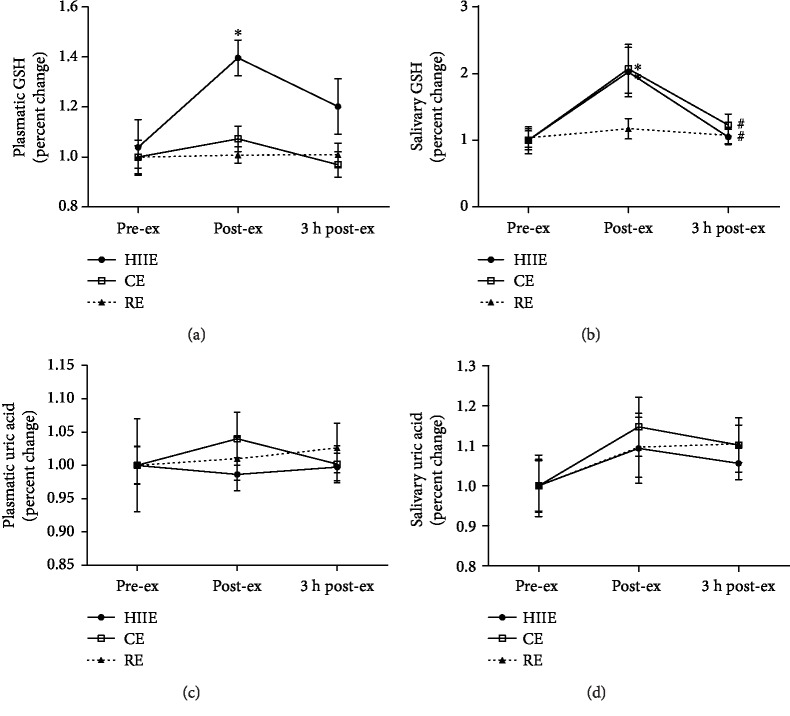
Nonenzymatic antioxidants: reduced glutathione (GSH) levels in plasma (a) and saliva (b); uric acid levels in plasma (c) and saliva (d), at preexercise, postexercise, and 3 hours postexercise. HIIE: high-intensity interval exercise; CE: continuous exercise; RE: resistance exercise. Values expressed as mean percentage in relation to preexercise ± SEM. ^∗^*p* ≤ 0.05 vs. pre-ex; ^#^*p* ≤ 0.05 vs. post-ex. ANOVA-RM followed by Tukey's test (*n* = 13).

Plasmatic and salivary antioxidants are shown in Figures 3–5. Total antioxidant capacity in plasma (FRAP) increased in all exercise protocols at post-ex compared to pre-ex. At 3 h post-ex, FRAP decreased in CE when compared to that at post-ex (Figure 3(a)). The antioxidant capacity in saliva samples showed similar results to that in plasma, with an increase in salivary FRAP in all exercise protocols at post-ex compared to pre-ex. At 3 h post-ex, salivary FRAP decreased in both CE and HIIE, and in RE, salivary antioxidant capacity remained increased (Figure 3(b)).

Plasmatic SOD activity decreased in the HIIE at post-ex compared to pre-ex. At 3 h post-ex, SOD activity increased compared to that at post-ex (Figure 4(a)). In the saliva samples, SOD activity decreased in HIIE and CE at post-ex compared to pre-ex and remained decreased in HIIE at 3 h post-ex (Figure 4(b)). Catalase activity in plasma increased in all exercise protocols at post-ex compared to pre-ex. At 3 h post ex, catalase activity decreased in both HIIE and CE when compared to that at post-ex (Figure 4(c)). Salivary catalase activity also increased in all exercise protocols at post-ex compared to pre-ex and remained elevated 3 hours after in the CE (Figure 4(d)).

GSH levels in plasma samples increased at post-ex compared to pre-ex in HIIE. No other changes of this biomarker were found in plasma (Figure 5(a)). In relation to salivary GSH levels, there was an increase in HIIE and CE at post-ex compared to pre-ex. At 3 h post-ex, salivary GSH decreased in both HIIE and CE when compared to that at post-ex (Figure 5(b)). No changes of GSH levels were observed in RE.

No differences were observed in uric acid concentrations in the plasma and saliva samples in none of the analyzed exercise protocols (Figures 5(c) and 5(d)).

Correlation between the antioxidants measured in plasma and saliva was evaluated at pre-ex, post-ex, and 3 h post-ex. At pre-ex, a strong positive correlation of total antioxidant capacity (FRAP) was observed between the plasma and saliva in the HIIE (*r* = 0.75, *p* = 0.003), CE (*r* = 0.76, *p* = 0.004), and RE (*r* = 0.87, *p* = 0.002) protocols. There was also a moderate and strong positive correlation of FRAP in the CE (*r* = 62, *p* = 0.03) and RE (*r* = 0.83, *p* = 0.003) at post-ex. 3 hours after exercise, a strong positive correlation between salivary and plasmatic FRAP was observed in the HIIE protocol (*r* = 0.74, *p* = 0.03). 3 h post-ex was also verified to have correlations in SOD activity in the HIIE and CE protocols (*r* = −0.62, *p* = 0.04, and *r* = −0.90, *p* = 0.0003, respectively) and uric acid in the CE protocol (*r* = 0.72, *p* = 0.007).

## 4. Discussion

In this study, we showed that different exercise protocols (HIIE: high-intensity interval exercise, CE: continuous exercise, and RE: resistance exercise) applied to trained individuals increased the levels of salivary intensity exercise markers (amylase activity and total protein), as well as the concentration of salivary nitric oxide. RE showed minor alterations in the antioxidants evaluated when compared to the other protocols, while HIIE and CE showed a more pronounced and similar antioxidant response profile. Interestingly, the antioxidant response in the saliva samples showed to be very similar to that in plasma, in all exercise protocols.

No differences were observed in the salivary flow comparing before and after exercise. Interestingly, data regarding the effects of exercise related to salivary flow are controversial. Some studies have found no effect of exercise over salivary flow [27, 28], whereas others have reported an increased salivary flux [29] and also a decreased flow [30] promoted by different protocols of exercise.

An increase in salivary alpha-amylase activity was observed after all exercise protocols (HIIE, CE, and RE). These results corroborate with other studies that also verified postexercise increase [31, 32], and such enzymatic behavior may occur due to the sympathetic stimulus promoted by exercise, with a direct effect on salivary glands by plasma catecholamines, such as norepinephrine [33].

An increase in salivary total protein after all the exercises (HIIE, CE, and RE) was observed, and these results are consistent with other studies that have also verified this increase after different physical exercise protocols [18, 31]. Taken together, the dosage of total protein along with amylase activity determination, as alternative methods to determine the intensity of an exercise, suggests here the similar and high load of the exercise protocols applied.

In relation to the effects of exercise on nitrite levels, it was observed that all protocols promoted its increase in the saliva samples. The dosage of salivary nitrite can be used as a representation of the production and total availability of nitric oxide by the body [34], and its evaluation is very important in sports medicine, since nitric oxide is strongly associated with the benefits of regular practice of physical exercise on hypertension and related diseases. This increase in nitric oxide bioavailability is attributed primarily to the increase in shear stress induced by physical exercise, which increases the production of nitric oxide and other vasorelaxant molecules, leading to a reduction in blood pressure [35].

Few studies have evaluated the acute effect of physical exercise on salivary nitrite levels in healthy, unsupplemented young individuals [13, 36]. Gonzalez et al. (2008) did not find differences in salivary nitrite levels after a continuous aerobic exercise protocol, whereas Rahman et al. (2010) observed its increase after an incremental test, with higher levels verified 1 hour after the exercise. Our results showed an increase in nitrite right after CE, whereas in HIIE and RE, this increase was verified after 3 hours after the end of the test. These data together with those of Rahman et al. (2010) suggest that the peak of salivary nitrite levels can occur hours after the exercise, pointing to the importance of nitrite monitoring at different moments for a better understanding about the effects of exercise on the modulation of nitric oxide levels. This increase in nitrite levels observed hours after the end of the exercise can also indicate the long-term benefits to the endothelial function that HIIE and RE could promote to the health.

The study of antioxidants and other biomarkers of oxidative stress induced by acute exercise in saliva samples is still poorly explored [12–14]. To our knowledge, only one study compared the response profile between plasma and saliva samples, where Deminice and collaborators (2010) suggest that saliva does not adequately reflect the plasma response. However, the study of Deminice and collaborators (2010) has some limitations; for example, only one type and protocol of exercise were evaluated (resistance) and no antioxidant enzyme was measured. Here, we analyzed the salivary and plasmatic antioxidants comparing different types of exercise protocols and investigated enzymatic and nonenzymatic parameters.

It is important to mention that there are some factors in the oral cavity that can be sources of salivary oxidative stress and therefore can alter results of the antioxidants measured. These factors include mainly the presence of periodontal inflammation, but also alcohol and cigarette consumption, ingestion of certain types of food, and dental treatment [37]. In this way, all necessary precautions were taken to avoid such samples.

Regarding the total antioxidant capacity, it was observed that the plasma and saliva samples showed a similar response profile, with an increase after all the exercise protocols (HIIE, CE, and RE). This result indicates the increase in antioxidant defenses in response to the probably oxidative stress induced by physical exercise and corroborates with the study by Gonzalez et al. (2008), who also showed an increase in salivary antioxidant capacity after a protocol of continuous aerobic exercise. Some authors [13, 38] attributed increased total antioxidant capacity mainly to uric acid levels. In this study, although there was an increase in uric acid levels, this change was not significant. In this way, we believe that this increase in total antioxidant capacity should be related to other components, such as high levels of GSH and other antioxidant molecules, not evaluated here.

A recent study compared the effect of different HIIE protocols on total plasma antioxidant capacity and found that all exercises increased antioxidant capacity at postexercise [39]. Our results show that only a single acute HIIE session is able to increase the total antioxidant capacity in saliva samples, similar to what occurs in plasma. In addition, the response profile in total antioxidant capacity observed in HIIE was similar to that observed in CE. An increase in plasma GSH levels was also observed after HIIE, whereas in saliva, GSH levels increased after HIIE and CE, and the RE showed no differences. The increase in plasma GSH followed by HIIE corroborates with another study that observed this increase after an HIIE protocol in swimmers [40] and shows the effect of these exercises on increasing the glutathione system of the antioxidant defense.

A decrease in SOD activity in plasma after HIIE and in saliva after HIIE and CE was verified. This decrease in SOD activity in plasma was also recently observed in a study that evaluated three different protocols: HIIE, sprint interval exercise, and continuous moderate-intensity exercise [41]. However, the mechanisms for such enzymatic behavior are not well elucidated. Here, we suggest that during the execution of the exercise, large amounts of H_2_O_2_ could have been generated (based on the observed high catalase activity), which may have promoted the inhibition of SOD and consequently decreased the activity observed in the postexercise. Some studies have already found this mechanism of inhibition of SOD promoted by H_2_O_2_*in vitro* [42, 43].

CAT activity increased in both plasma and saliva samples after all exercise protocols (HIIE, CE, and RE). This result corroborates with other studies that have also verified this increase at postexercise [12, 41]. Such increase in CAT activity may be due to the increased release of this enzyme by muscle cells and erythrocytes promoted by exercise [44], showing again the effect of the applied protocols on the increase in the antioxidant system. There were no differences in uric acid levels, suggesting that other antioxidant molecules are acting as a defense mechanism against the presupposed oxidative stress induced by exercise.

Michailidis et al. (2007) verified that plasmatic antioxidants and other oxidative stress biomarkers showed peaks and returned to the baseline at specific and different times after an aerobic exercise session, making it difficult to establish a better time for biomarker analysis. Here, the analyzed parameters showed different recovery times according to the exercise protocol applied and also according to the biological fluid studied. However, the recovery period of 3 hours proposed here allowed verifying the return to baseline of the majority of biomarkers, such as total protein, total antioxidant capacity, GSH, and SOD, CAT, and amylase activities.

Correlation analysis between antioxidants in plasma and saliva samples at pre-, post-, and 3 h postexercise was generally inconsistent, with a correlation pattern only observed in the total antioxidant capacity evaluated by the FRAP method. Interestingly, no correlation pattern was found in the other antioxidants analyzed, even though the response of saliva parameters seemed to be very similar to that of plasma.

The RE protocol used in this study, unlike HIIE and CE, showed no changes in SOD activity and GSH concentrations. Probably, the generation of ROS promoted by this type of exercise was smaller when compared to the others. Shi et al., when evaluating aerobic and anaerobic exercises with similar workloads, suggested that aerobic exercise initially seems to generate more ROS, whereas anaerobic exercise may induce a longer generation of these species [22]. Also, these minor changes in the antioxidant parameters observed in the RE may happen due to the less muscle perfusion in this group, compared with HIIE and CE. Our study showed that a single HIIE session exerts a pattern of antioxidant response very similar to a CE session, verified in both plasma and saliva fluids. The mechanisms by which interval exercise presented similar long-term continuous outcomes may be related to increased mechanical stress promoted in the skeletal muscle [45] and also by the increase in metabolic fluctuations induced by this type of exercise [46].

Limitations of this study include the small number of individuals evaluated and the lack of final oxidative stress biomarkers, such as lipid peroxides and damage to DNA, which may be investigated in future studies.

## 5. Conclusions

This is the first study that evaluated salivary and plasmatic antioxidants comparing the present exercises. Overall, the antioxidant response profile of the saliva samples showed to be very similar to that of plasma, in all exercise protocols: HIIE, CE, and RE. Thus, our results suggest that the collection and analysis of saliva samples may be an alternative tool for the study of antioxidants in different protocols of physical exercise. This is quite interesting, since saliva demands a noninvasive collection method, presenting as a more feasible procedure for application in future athlete studies and others that require a greater number of collections, although it is worth mentioning that correlation analysis between antioxidants in plasma and saliva samples was generally inconsistent. Moreover, since the HIIE session exerted a similar pattern of antioxidant response when compared to CE, in saliva and plasma samples, we can also suggest that HIIE may lead to alterations in antioxidants and consequently to the physiological processes related to redox, similar to the CE, with the advantage of being performed in a shorter time.

## Figures and Tables

**Table 1:** Salivary flow (mL min^−1^) at preexercise, postexercise, and 3 hours postexercise. HIIE: high-intensity interval exercise; CE: continuous exercise; RE: resistance exercise.

	Pre-ex	Post-ex	3 h post-ex
HIIE	0.97 ± 0.11	0.80 ± 0.11	1.07 ± 0.13^#^
CE	0.95 ± 0.11	0.81 ± 0.08	1.12 ± 0.08^#^
RE	0.98 ± 0.14	0.91 ± 0.12	1.08 ± 0.13

Values expressed as mL min^−1^ ± SEM. ^∗^*p* ≤ 0.05 vs. pre-ex; ^#^*p* ≤ 0.05 vs. post-ex. ANOVA-RM followed by Tukey's test (*n* = 13).

## Data Availability

The data used to support the findings of this study are included within the article.

## References

[1] Finkler M., Lichtenberg D., Pinchuk I. (2014). The relationship between oxidative stress and exercise. *Journal of Basic and Clinical Physiology and Pharmacology*.

[2] Sies H. (1997). Oxidative stress: oxidants and antioxidants. *Experimental Physiology*.

[3] Ji L. L., Gomez-Cabrera M. C., Vina J. (2006). Exercise and hormesis: activation of cellular antioxidant signaling pathway. *Annals of the New York Academy of Sciences*.

[4] Sen C. K. (2001). Antioxidant and redox regulation of cellular signaling: introduction. *Medicine and Science in Sports and Exercise*.

[5] Radak Z., Taylor A. W., Ohno H., Goto S. (2001). Adaptation to exercise-induced oxidative stress: from muscle to brain. *Exercise Immunology Review*.

[6] Sjodin B., Hellsten Westing Y., Apple F. S. (1990). Biochemical mechanisms for oxygen free radical formation during exercise. *Sports Medicine*.

[7] Powers S. K., Jackson M. J. (2008). Exercise-induced oxidative stress: cellular mechanisms and impact on muscle force production. *Physiological Reviews*.

[8] Ziemann E., Grzywacz T., Luszczyk M., Laskowski R., Olek R. A., Gibson A. L. (2011). Aerobic and anaerobic changes with high-intensity interval training in active college-aged men. *Journal of Strength and Conditioning Research*.

[9] Gibala M. J., Little J. P., Macdonald M. J., Hawley J. A. (2012). Physiological adaptations to low-volume, high-intensity interval training in health and disease. *The Journal of Physiology*.

[10] Stutts W. C. (2002). Physical activity determinants in adults. Perceived benefits, barriers, and self efficacy. *AAOHN Journal*.

[11] Bartlett J. D., Close G. L., MacLaren D. P., Gregson W., Drust B., Morton J. P. (2011). High-intensity interval running is perceived to be more enjoyable than moderate-intensity continuous exercise: implications for exercise adherence. *Journal of Sports Sciences*.

[12] Cavas L., Arpinar P., Yurdakoc K. (2005). Possible interactions between antioxidant enzymes and free sialic acids in saliva: a preliminary study on elite judoists. *International Journal of Sports Medicine*.

[13] Gonzalez D., Marquina R., Rondon N., Rodriguez-Malaver A. J., Reyes R. (2008). Effects of aerobic exercise on uric acid, total antioxidant activity, oxidative stress, and nitric oxide in human saliva. *Research in sports medicine (Print)*.

[14] Deminice R., Sicchieri T., Payao P. O., Jordao A. A. (2010). Blood and salivary oxidative stress biomarkers following an acute session of resistance exercise in humans. *International Journal of Sports Medicine*.

[15] Malamud D. (2011). Saliva as a diagnostic fluid. *Dental Clinics of North America*.

[16] Hayes L. D., Grace F. M., Baker J. S., Sculthorpe N. (2015). Exercise-induced responses in salivary testosterone, cortisol, and their ratios in men: a meta-analysis. *Sports Medicine*.

[17] Diaz M. M., Bocanegra O. L., Teixeira R. R., Soares S. S., Espindola F. S. (2013). Salivary nitric oxide and alpha-amylase as indexes of training intensity and load. *International Journal of Sports Medicine*.

[18] Bortolini M. J., De Agostini G. G., Reis I. T., Lamounier R. P., Blumberg J. B., Espindola F. S. (2009). Total protein of whole saliva as a biomarker of anaerobic threshold. *Research Quarterly for Exercise and Sport*.

[19] de Oliveira V. N., Bessa A., Lamounier R. P., de Santana M. G., de Mello M. T., Espindola F. S. (2010). Changes in the salivary biomarkers induced by an effort test. *International Journal of Sports Medicine*.

[20] Leaf D. A., Kleinman M. T., Hamilton M., Barstow T. J. (1997). The effect of exercise intensity on lipid peroxidation. *Medicine and Science in Sports and Exercise*.

[21] Bloomer R. J., Davis P. G., Consitt L. A., Wideman L. (2007). Plasma protein carbonyl response to increasing exercise duration in aerobically trained men and women. *International Journal of Sports Medicine*.

[22] Shi M., Wang X., Yamanaka T., Ogita F., Nakatani K., Takeuchi T. (2007). Effects of anaerobic exercise and aerobic exercise on biomarkers of oxidative stress. *Environmental Health and Preventive Medicine*.

[23] Borg G. A. (1974). Perceived exertion. *Exercise and Sport Sciences Reviews*.

[24] Bradford M. M. (1976). A rapid and sensitive method for the quantitation of microgram quantities of protein utilizing the principle of protein-dye binding. *Analytical Biochemistry*.

[25] Teixeira R. R., de Souza A. V., Peixoto L. G. (2017). Royal jelly decreases corticosterone levels and improves the brain antioxidant system in restraint and cold stressed rats. *Neuroscience Letters*.

[26] Justino A. B., Pereira M. N., Peixoto L. G. (2017). Hepatoprotective properties of a polyphenol-enriched fraction fromAnnona crassifloraMart. fruit peel against diabetes-induced oxidative and nitrosative stress. *Journal of Agricultural and Food Chemistry*.

[27] Pilardeau P., Richalet J. P., Bouissou P., Vaysse J., Larmignat P., Boom A. (1990). Saliva flow and composition in humans exposed to acute altitude hypoxia. *European Journal of Applied Physiology and Occupational Physiology*.

[28] Dawes C. (1981). The effects of exercise on protein and electrolyte secretion in parotid saliva. *The Journal of Physiology*.

[29] Bardon A., Ceder O., Kollberg H. (1983). Cystic fibrosis-like changes in saliva of healthy persons subjected to anaerobic exercise. *Clinica Chimica Acta*.

[30] Sari-Sarraf V., Reilly T., Doran D. A., Atkinson G. (2007). The effects of single and repeated bouts of soccer-specific exercise on salivary IgA. *Archives of Oral Biology*.

[31] Walsh N. P., Blannin A. K., Clark A. M., Cook L., Robson P. J., Gleeson M. (1999). The effects of high-intensity intermittent exercise on saliva IgA, total protein and alpha-amylase. *Journal of Sports Sciences*.

[32] Li T. L., Gleeson M. (2004). The effect of single and repeated bouts of prolonged cycling and circadian variation on saliva flow rate, immunoglobulin A and alpha-amylase responses. *Journal of Sports Sciences*.

[33] Chatterton R. T., Vogelsong K. M., Lu Y. C., Ellman A. B., Hudgens G. A. (1996). Salivary *α*-amylase as a measure of endogenous adrenergic activity. *Clinical Physiology*.

[34] Bryan N. S. (2015). The potential use of salivary nitrite as a marker of NO status in humans. *Nitric Oxide*.

[35] Kingwell B. A. (2000). Nitric oxide-mediated metabolic regulation during exercise: effects of training in health and cardiovascular disease. *The FASEB Journal*.

[36] Rahman Z. A., Abdullah N., Singh R., Sosroseno W. (2010). Effect of acute exercise on the levels of salivary cortisol, tumor necrosis factor-alpha and nitric oxide. *Journal of Oral Science*.

[37] Zukowski P., Maciejczyk M., Waszkiel D. (2018). Sources of free radicals and oxidative stress in the oral cavity. *Archives of Oral Biology*.

[38] Child R. B., Wilkinson D. M., Fallowfield J. L., Donnelly A. E. (1998). Elevated serum antioxidant capacity and plasma malondialdehyde concentration in response to a simulated half-marathon run. *Medicine and Science in Sports and Exercise*.

[39] Cipryan L. (2017). IL-6, antioxidant capacity and muscle damage markers following high-intensity interval training protocols. *Journal of Human Kinetics*.

[40] Deminice R., Trindade C. S., Degiovanni G. C. (2010). Oxidative stress biomarkers response to high intensity interval training and relation to performance in competitive swimmers. *The Journal of Sports Medicine and Physical Fitness*.

[41] Parker L., Trewin A., Levinger I., Shaw C. S., Stepto N. K. (2017). Exercise-intensity dependent alterations in plasma redox status do not reflect skeletal muscle redox-sensitive protein signaling. *Journal of Science and Medicine in Sport*.

[42] Bray R. C., Cockle S. A., Fielden E. M., Roberts P. B., Rotilio G., Calabrese L. (1974). Reduction and inactivation of superoxide dismutase by hydrogen peroxide. *Biochemical Journal*.

[43] Goldstone A. B., Liochev S. I., Fridovich I. (2006). Inactivation of copper, zinc superoxide dismutase by H_2_O_2_ : mechanism of protection. *Free Radical Biology & Medicine*.

[44] Michailidis Y., Jamurtas A. Z., Nikolaidis M. G. (2007). Sampling time is crucial for measurement of aerobic exercise-induced oxidative stress. *Medicine and Science in Sports and Exercise*.

[45] Fisher-Wellman K., Bloomer R. J. (2009). Acute exercise and oxidative stress: a 30 year history. *Dynamic Medicine*.

[46] Combes A., Dekerle J., Webborn N., Watt P., Bougault V., Daussin F. N. (2015). Exercise-induced metabolic fluctuations influence AMPK, p38-MAPK and CaMKII phosphorylation in human skeletal muscle. *Physiological Reports*.

